# Methotrexate-Induced Apoptotic Colopathy in Crohn's Disease

**DOI:** 10.14309/crj.0000000000000980

**Published:** 2023-02-23

**Authors:** Vibhu Chittajallu, Wendy Liu, Vu Nguyen

**Affiliations:** 1 Division of Gastroenterology and Liver Disease, University Hospitals Cleveland Medical Center, Cleveland, OH; 2 Division of Clinical Pathology, University Hospitals Cleveland Medical Center, Cleveland, OH

**Keywords:** Crohn's disease, methotrexate, pathology

## Abstract

Apoptotic colopathy is a pattern of injury not frequently associated with Crohn's disease (CD) despite its association with medications used in CD management. We describe a patient with CD on methotrexate who underwent a diagnostic colonoscopy for abdominal pain and diarrhea, with biopsies demonstrating apoptotic colopathy. After methotrexate discontinuation, repeat colonoscopy demonstrated resolution of apoptotic colopathy in addition to diarrhea improvement.

## INTRODUCTION

Histology of Crohn's disease (CD) is characterized by patchy transmural inflammatory pattern, noncaseating epithelioid granulomas, and crypt architectural distortion; however, apoptotic colopathy is not typically seen.^1^ Apoptotic colopathy is a pattern of injury featuring predominantly crypt epithelial apoptosis and can be seen secondary to drug-induced injury or viral infections.^2^ We present a patient with active CD with concurrent apoptotic colopathy with resolution after methotrexate discontinuation.

## CASE REPORT

**Figure 1.**
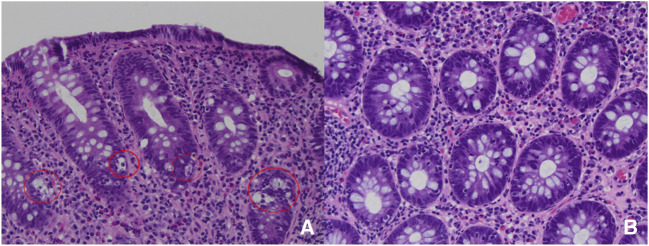
(A) Left colon biopsy on methotrexate with apoptotic bodies marked. (B) Left colon biopsy off methotrexate with resolution of apoptotic bodies.

A 35-year-old woman with a history of Crohn's colitis with perianal disease on medical therapy of ustekinumab every 4 weeks and methotrexate 25 mg weekly for the past year after failing multiple prior biologics underwent colonoscopy for abdominal pain and diarrhea evaluation. Colonoscopy demonstrated recurrent anal stenosis, segmental inflammation with ulcerations in the left colon, and otherwise normal-appearing right colon and terminal ileum. Biopsies of the left colon demonstrated preserved crypt architecture with expansion of the lamina propria by a mixed inflammatory infiltrate plus cryptitis and reactive epithelial changes. Numerous apoptotic bodies were seen primarily in the crypt basal portions and were the predominant lesions noted in the left colon biopsies (Figure 1). Cytomegalovirus stain was negative. A diagnosis of mild focal active colitis and apoptotic colopathy, possibly drug-related, was determined. Methotrexate was suspended. In addition, infliximab was added to ustekinumab for CD management. The patient noted improvement in her abdominal pain and diarrheal symptoms.

Repeat colonoscopy 4 months later demonstrated recurrent anal stenosis, but no active mucosal inflammation. Biopsies of the left colon showed persistent inflammatory cells in the lamina propria but complete resolution of previous predominant apoptotic bodies (Figure 1).

## DISCUSSION

Apoptotic colopathy is not a typical histologic feature seen in association with CD. Medications associated with this pattern of colonic injury are primarily immunosuppressants: mycophenolate mofetil, CTLA-4 inhibitors (ipilimumab), anti-PD-1 (nivolumab), idelalisib, tumor necrosis alpha inhibitors (etanercept, infliximab), and antimetabolites (methotrexate, capecitabine).^2^ Patients with apoptotic colopathy typically present with watery diarrhea that resolves with cessation of these medications.^2^

Our patient demonstrated a rare side effect of methotrexate in the setting of active CD. The patient had improvement in her gastrointestinal symptoms with cessation of methotrexate and resolution of apoptotic bodies on repeat evaluation; however, her biologic therapies were also escalated. To the best of our knowledge, this is the first histologic description of methotrexate-induced apoptotic colopathy in a patient with CD.

## DISCLOSURES

Author contributions: V. Chittajallu: contribution to concept and drafting work and is the article guarantor. W. Liu: interpretation of data. V. Nguyen: contribution to concept and final approval.

Financial disclosure: None to report.

Informed consent was obtained for this case report.
